# Strong Genome-Wide Selection Early in the Evolution of *Prochlorococcus* Resulted in a Reduced Genome through the Loss of a Large Number of Small Effect Genes

**DOI:** 10.1371/journal.pone.0088837

**Published:** 2014-03-03

**Authors:** Zhiyi Sun, Jeffrey L. Blanchard

**Affiliations:** 1 Graduate Program in Organismic and Evolutionary Biology, University of Massachusetts, Amherst, Massachusetts, United States of America; 2 Department of Biology, University of Massachusetts, Amherst, Massachusetts, United States of America; British Columbia Centre for Excellence in HIV/AIDS, Canada

## Abstract

The smallest genomes of any photosynthetic organisms are found in a group of free-living marine cyanobacteria, *Prochlorococcus*. To determine the underlying evolutionary mechanisms, we developed a new method to reconstruct the steps leading to the *Prochlorococcus* genome reduction using 12 *Prochlorococcus* and 6 marine *Synechococcus* genomes. Our results reveal that small genome sizes within *Prochlorococcus* were largely determined shortly after the split of *Prochlorococcus* and *Synechococcus* (an early big shrink) and thus for the first time decouple the genome reduction from *Prochlorococcus* diversification. A maximum likelihood approach was then used to estimate changes of nucleotide substitution rate and selection strength along *Prochlorococcus* evolution in a phylogenetic framework. Strong genome wide purifying selection was associated with the loss of many genes in the early evolutionary stage. The deleted genes were distributed around the genome, participated in many different functional categories and in general had been under relaxed selection pressure. We propose that shortly after *Prochlorococcus* diverged from its common ancestor with marine *Synechococcus*, its population size increased quickly thus increasing efficacy of selection. Due to limited nutrients and a relatively constant environment, selection favored a streamlined genome for maximum economy. Strong genome wide selection subsequently caused the loss of genes with small functional effect including the loss of some DNA repair genes. In summary, genome reduction in *Prochlorococcus* resulted in genome features that are similar to symbiotic bacteria and pathogens, however, the small genome sizes resulted from an increase in genome wide selection rather than a consequence of a reduced ecological niche or relaxed selection due to genetic drift.

## Introduction

Genome sizes vary in bacteria, ranging from 160 kb in *Carsonella ruddii* [1] to 13,034 kb in *Sorangium cellulosum* [2]. In general, bacterial genomes are tightly packed with coding genes and the gene length is similar across genomes [3], [4]. Therefore, differences in the bacterial genome sizes are primarily the result of loss of existing genes and acquiring new genes through the processes of gene duplication and horizontal gene transfer.

There are three well-known examples of bacteria with reduced genomes: (1) pathogens (e.g., *Mycoplasma genitalium* [5] and *Rickettsia* [6]), (2) endosymbionts (e.g., *Buchnera* [7], *Wigglesworthia* [8] and *Blochmannia* [9]), and (3) marine microbes that play important ecological roles in oligotrophic environments (e.g., *Pelagibacter ubique* [10] and *Prochlorococcus marinus* [11]). Understanding genome reduction is important for understanding the evolution of human pathogens and agricultural pests. It also helps us to gain insights into global nutrient cycling and climate regulation.

Reduced genomes in obligate pathogens are associated with a change from a free-living to a host-dependent lifestyle [12]. This lifestyle change rendered many genes unnecessary for the organisms' survival in the protected environments [13], [14]. Moreover, due to the small effective population sizes and reduced recombination, the effect of genetic drift is larger. Genetic drift facilitates fixation of small effect deleterious mutations, formation of pseudogene, and subsequent gene deletions [12], [15]. In the cases of endosymbiotic bacteria, the host environments restrict opportunities of gene gains through horizontal gene transfer [16]–[18]. Although pathogens can acquire genes via lateral gene transfers, it has been demonstrated that obligate parasite genomes are subject to far more extensive DNA loss due to inactivation of unnecessary genes and deletional bias [6], [13]. It is suggested that genome degradation in obligate host-associated bacteria is so advanced that it has placed them on the path to extinction [19], [20].

The marine cyanobacteria in the *Prochlorococcus* genus are the most abundant known oxygenic phototrophs on earth, and contribute significantly to the primary productivity in the ocean [21]. *Prochlorococcus* genomes are also among the smallest of all oxygen-evolving autotrophs. Most of the sequenced *Prochlorococcus* genomes, ranging in size from 1.6 to 2.7 Mega-base pairs (http://www.ncbi.nlm.nih.gov/genome), are smaller than those of another major marine picocyanobacterial genus *Synechococcus*, which is the closest phylogenetic group to *Prochlorococcus* [21]. These two groups differ in light-harvesting apparatus and nutrient utilization, and thus have quite different ecology performance [21]. Although *Prochlorococcus* and *Synechococcus* commonly coexist, *Prochlorococcus* has wider vertical distribution in oceanic waters relative to *Synechococcus* and dominates in the oligotrophic regions of the ocean [21], [22]. In contrast, *Synechococcus* is much more abundant in nutrient rich environments such as upwelling areas and coastal watersheds [23]. *Prochlorococcus* isolates can be divided into two major ecotypes exhibiting distinct light and/or temperature optima: the high light-adapted (HL) and low light-adapted (LL) clades [24], [25]. Aligned with the ecological distinction, genome sizes vary between the *Prochlorococcus* ecotypes that the HL genomes are smaller than most of the LL genomes [26]. Previous studies have revealed evidence for genome reduction in *Prochlorococcus* [11], [26]–[30]. In contrast, no extensive genome streamlining has occurred during the evolution of marine *Synechococcus* [31].

Reduced *Prochlorococcus* genomes share many genomic characteristics with the aforementioned endosymbiotic bacteria such as an increase in AT content, loss of DNA repair genes and accelerated evolution [27], [29], [32]. However, in sharp contrast to small population obligate host-associated bacteria, *Prochlorococcus* is possibly the most plentiful free-living bacterial genus on Earth, and the sizes of *Prochlorococcus* populations are vast [11], [25], [27], [33]. Moreover, purifying selection is estimated to be much stronger in the present-day *Prochlorococcus populations* than in the marine *Synechococcus* [32]. There is also evidence suggesting that *Prochlorococcus* has experienced a fair level of genetic exchange via interactions with different types of phages [34]–[36]. Therefore, the evolutionary forces that shaped the small genomes of *Prochlorococcus* are probably different from the forces that caused the genome reduction of endosymbionts and pathogens [27]. It is also intriguing why a free-living, ecologically successful organism would become smaller.

Several hypotheses have been proposed to explain genome reduction in free-living bacteria. According to the genome-streamlining hypothesis [10], small size, therefore larger surface-to-volume ratio, gives the organisms a selective advantage in nutrient poor water. It's also possible that with deletions of non-essential genes, *Prochlorococcus* have adopted a more economical lifestyle to better cope with the nutrient limited environment [27]. The “Black Queen Hypothesis” suggests that vital but costly genes could be deleted under strong purifying selection if co-occurring organisms can provide the lost functions [37]. Alternatively, an accelerated protein evolution rate was observed in the small genomes of *Prochlorococcus*, and this led to the suggestion that an elevated genome-wide mutation rate is associated with genome reduction [27]. Another study [38] demonstrated through mathematical modeling and computer simulation that a modest increase in mutation rate could lead to significant genome reduction even in an organism having a very large population size, thus creating the possibility that enhanced mutation rate, as a non-selective force, could be the driving force of genome reduction in free-living bacteria. However, no study has systemically examined the effects of natural selection and molecular evolution rate on the genome content of *Prochlorococcus* in the evolutionary history.

In this study, we analyzed the complete genome sequences of 12 *Prochlorococcus* isolates and 6 marine *Synechococcus* isolates to address the following questions: When and how did genome reduction occur in the evolutionary history of *Prochlorococcus*? Which genes were deleted from the *Prochlorococcus* genomes, and what are their functional effects? What role has natural selection played in *Prochlorococcus* genome evolution, particularly in the genome reduction? What is the driving force behind the reduced genomes of *Prochlorococcus*?

## Results

### Reconstruct Phylogeny of *Prochlorococcus* and *Synechococcus* using concatenated protein alignments

Based on a published phylogeny of marine cyanobacteria [28], 12 *Prochlorococcus* isolates (*P.*MED4, *P.*MIT9515, *P.*AS901, *P.*MIT9301, *P.*MIT9215, *P.*MIT9312, *P.*NATL1A, *P.*NATL2A, *P.*SS120, *P.*MIT9215, *P.*MIT9313, *P.*MIT9303) formed a monophyletic group and 5 marine *Synechococcus* isolates (*S.*CC9605, *S.*CC9902, *S.*WH8102, *S.*CC9311, *S.*WH7803) are in the sister group to *Prochlorococcus*. We used the above 17 genomes as the primary study material and refer to them as in-group genomes. *Synechococcus* RCC307 was used as an out-group.

**Figure 1:**
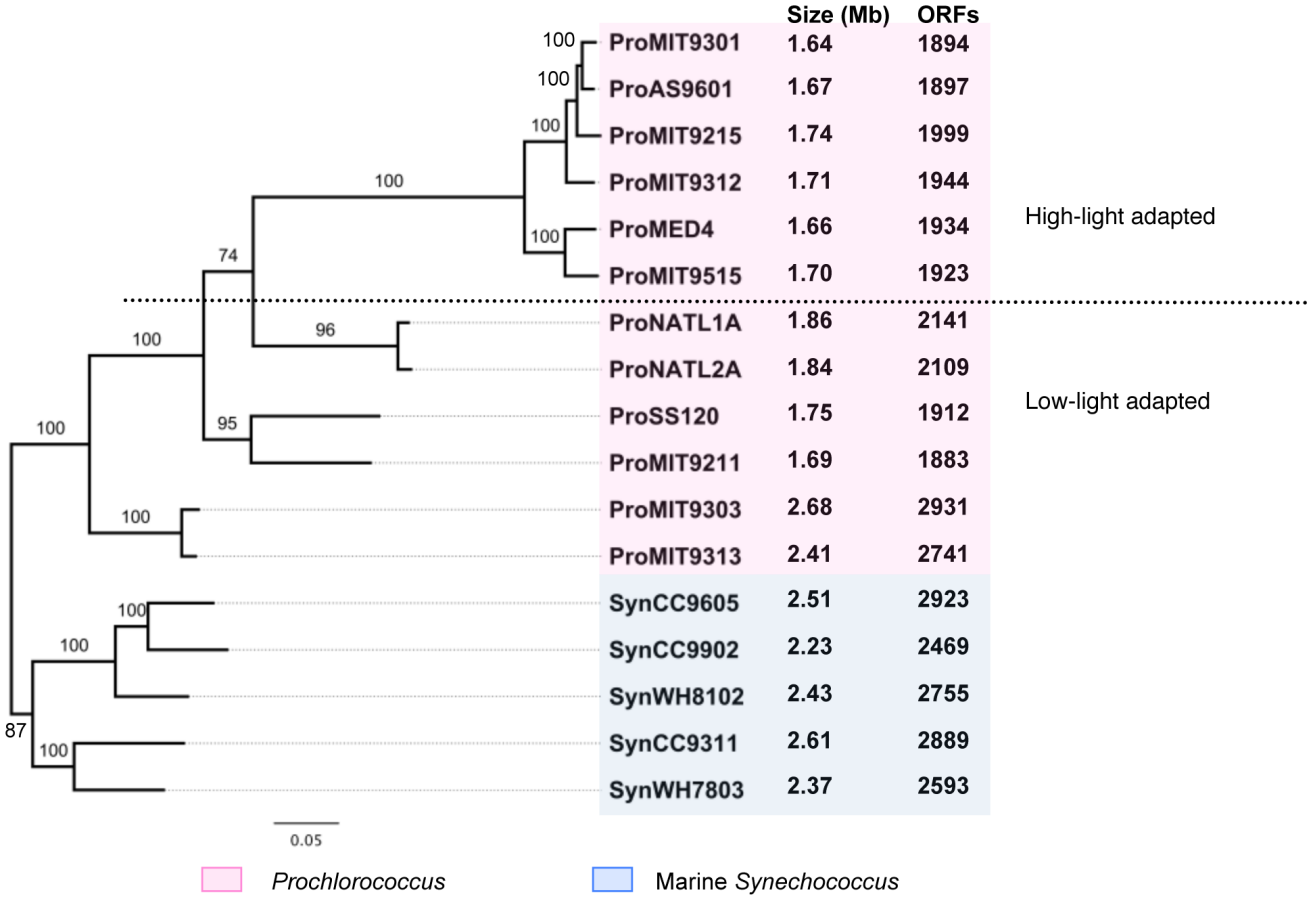
Phylogeny of *Prochlorococcus* and marine *Synechococcus* with genome sizes and protein-coding gene counts. The phylogenetic relationship of 12 *Prochlorococcus* and 5 marine *Synechococcus* is reconstructed from concatenation of 100 conserved protein alignments using a maximum likelihood analysis with Tree-PUZZLE implemented in PHYLIP 3.6a. The branch length is defined as the expected number of amino acid substitutions per site. Support for the internal branches of the quartet puzzling tree topology is shown as a percentage. Genome size and gene count are reported beside each genome.

An accurate phylogeny is critical for ancestral gene content reconstruction. Therefore, the first thing we did was to reconstruct the phylogeny of the 17 in-group genomes based on a concatenated alignment containing 100 of the most conserved orthologs. In brief, we first identified 1102 consistent orthologs from the 17 in-group genomes (see Materials and Methods). We then calculated the synonymous and non-synonymous substitution rates for every ortholog pair. Orthologs that have low non-synonymous substitution rate to synonymous substitution rate ratios, i.e., *dN*/*dS* ratio, are considered evolutionary conserved genes. Therefore, we selected 100 genes that have the smallest mean *dN*/*dS* ratios and moderate synonymous substitution rates (in the range of the 1^st^ and the 3^rd^ quartiles). Their protein sequences were aligned individually, and the alignments were concatenated. Finally a maximum likelihood tree was built using the combined information of the 100 orthologous genes. Our tree (Fig. 1) has the same topology as the previously reported trees [26], [28], [31]. In addition, it has higher bootstrap values for the internal nodes. Therefore, we are very confident that we have a reliable and robust phylogenetic framework for the analyses described below.

### Ancestral gene content reconstruction suggests early genome reduction in *Prochlorococcus* evolution

The sizes of *Prochlorococcus* genomes are generally smaller than those of the marine *Synechococcus* except for MIT9303 and MIT9313 (Fig. 1). Regression analysis result indicates a strong correlation (R^2^ = 0.995) between the genome size and the gene number of *Prochlorococcus* and *Synechococcus*. Also, the gene length does not significantly differ between the *Synechococcus* group and the *Prochlorococcus* group (Mann-Whitney U test, *P-*value = 0.959). This confirms that reduced gene number is the major cause of genome size reduction in *Prochlorococcus*. Among *Prochlorococcus*, there exist several distinct genome size groups in some accordance with the phylogeny (Fig. 1). Members in the low-light-adapted (LL) *P*. MIT9303 clade (MIT9303 and MIT9313) have the largest genome sizes among all the *Prochlorococcus* and they were the first to diverge from the other *Prochlorococcus* isolates. In contrast, members in a more recent LL clade *P.* NATL clade (NATL1A and NATL2A) have smaller genome sizes. Moreover, all the members of the youngest *Prochlorococcus* clade, i.e., the high-light-adapted (HL) clade (MED4, MIT9515, AS9601, MIT9301, MIT9215 and MIT9312), have the smallest genome sizes of *Prochlorococcus*. Interestingly, two LL *Prochlorococcus* isolates SS120 and MIT9211, which are relatively deep in the tree, have small genome sizes that are comparable to the HL clade (Fig. 1).

The next question we address is when and how did genome reduction happen? More specifically, is it a progressive and ongoing process, or was it a historical event? To address this question, we reconstructed the ancestral gene content at every node of the tree using two methods: maximum parsimony and maximum likelihood (See Methods and Materials). The results of these two approaches have led to the same conclusion of *Prochlorococcus* genome size evolution, and hence we will only report the results of the parsimony reconstruction.

The reconstructed last common ancestor of the 17 in-group genomes contains a total of 2028 genes. We first calculated the net gene losses and gains in the individual genomes from the last common ancestor (Table 1). The t-statistic of a Student's t test shows that *Prochlorococcus* lost significantly more genes than the *Synechococcus* (*P*<10^−6^) on average. Even the two largest *Prochlorococcus* genomes (MIT9303 and MIT9313) have significantly greater gene loss than *Synechococcus* (*P*<0.001). In contrast, while relatively large *Prochlorococcus* genomes (e.g., the moderate-size LL *P.* NATL clade and the large-size *P.* MIT9303 clade) have gained considerably more genes than the small genomes in the HL clade, the difference in gene gains between the small genomes in the LL *P.* SS120 clade and the HL clade is insignificant (Table 1).

**Table 1:** Summary of ORFs and genome fluxes of the 17 in-group marine cyanobacteria.

Strains	Total ORFs	Core genes	Genes in the last common ancestor	Total gene loss	Total gene gain
P.MED4	1897	1186	2028	608	364
P.MIT9515	1894	1186	2028	631	372
P.MIT9301	1944	1186	2028	605	343
P.AS9601	1999	1186	2028	594	343
P.MIT9215	1934	1186	2028	602	400
P.MIT9312	1923	1186	2028	594	365
P.NATL1A	2141	1186	2028	555	478
P.NATL2A	2109	1186	2028	564	456
P.SS120	1883	1186	2028	581	327
P.MIT9211	1912	1186	2028	576	297
P.MIT9303	2931	1186	2028	293	857
P.MIT9313	2741	1186	2028	298	750
S.CC9605	2923	1186	2028	129	708
S.CC9902	2469	1186	2028	189	407
S.WH8102	2755	1186	2028	125	546
S.CC9311	2889	1186	2028	133	685
S.WH7803	2593	1186	2028	120	426

**Figure 2:**
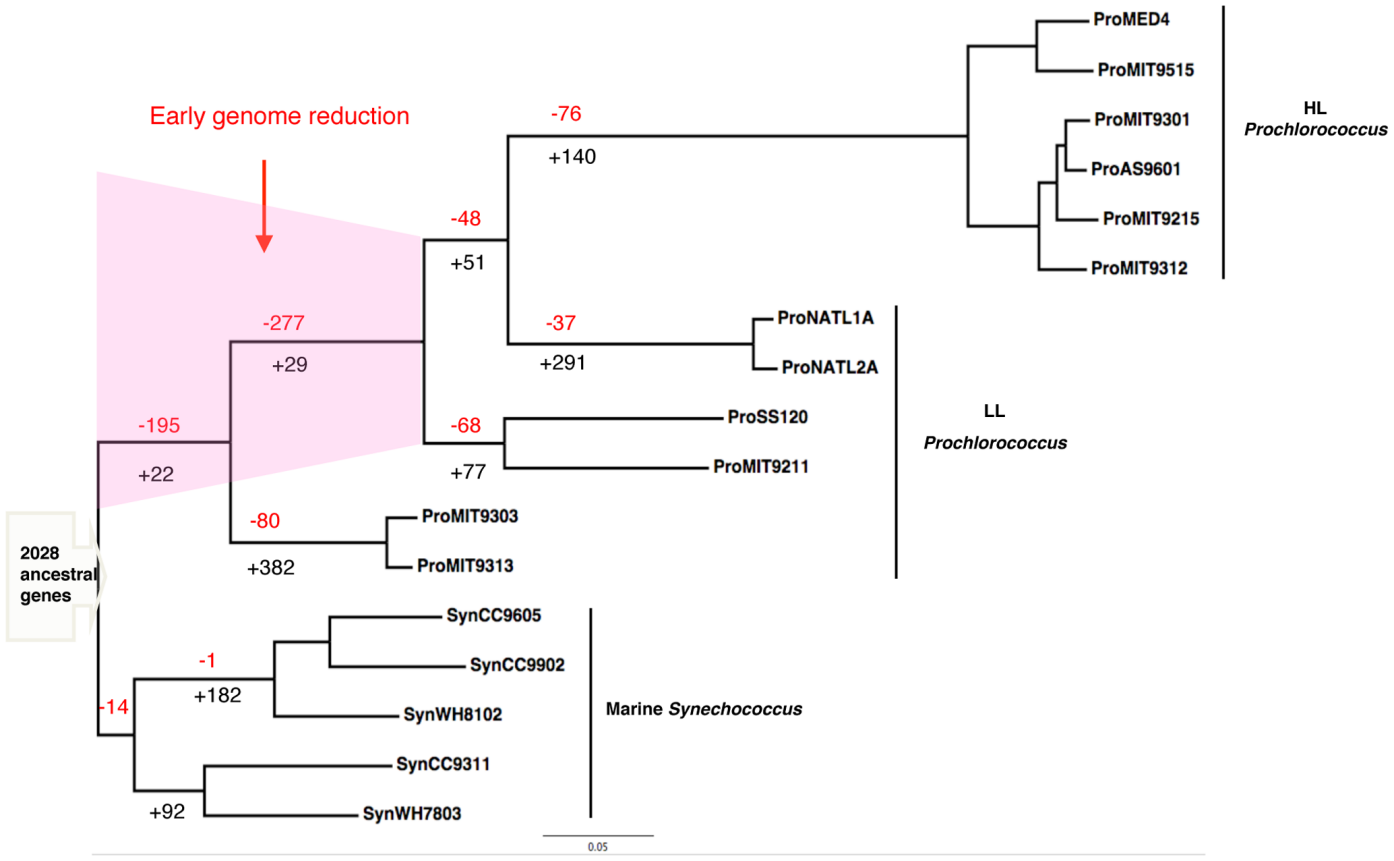
Summary of gene loss and gene gain events across the phylogenetic tree. Ancestral genome contents were reconstructed based on the phylogenetic tree in Figure 1 by maximum parsimony. Gene loss and gain events are summarized on the branches leading to the major clades of *Prochlorococcus*. The number in black is gene gain and the number in red is gene loss.

A more detailed investigation of gene loss and gene gain at every branch of the tree found that in spite of the fact that gene loss occurred at every stage of *Prochlorococcus* evolution, a significant amount of genes were deleted shortly after the split of *Prochlorococcus* and *Synechococcus*, and only a small number of genes were acquired during the same period (Fig. 2). Therefore, a reduced genome was established before the divergence of the HL clade and the LL clade. After the divergence from the other *Prochlorococcus*, both of the intermediate-size *P.* NATL clade and the large-size *P.* MIT9303 clade obtained a considerable number of genes, contributing significantly to their present-day relatively large genome sizes. Our results suggest that the observed distinct genome size groups within *Prochlorococcus* were shaped by a combination of an early and large genome reduction and clade-specific gene gains at late stages, instead of by progressive gene deletions.

### Intensified purifying selection associated with genome size reduction of *Prochlorococcus*

To determine the effects of natural selection on the genome evolution of *Prochlorococcus*, we carried out a maximum likelihood analysis to estimate the ratio of non-synonymous substitution rate to synonymous substitution rate, i.e., the *dN/dS* ratio, across the phylogenetic tree. The *dN/dS* ratio is used as an indicator of selective pressure acting on protein-coding genes. In this study, the substitutions rates were calculated from a concatenated alignment of 1102 consistent orthologs (see Materials and Methods). In addition to the 12 *Prochlorococcus*, 2 marine *Synechococcus* WH8102 and WH7803 each representing a *Synechococcus* clade, and an out-group marine *Synechococcus* RCC307 were used in the analysis.

Using the codeml program in the Phylogenetic Analysis by the Maximum Likelihood (PAML) package [39], we first applied a one-ratio model for the entire tree. This resulted in a log likelihood value of *l_1_* = −9300717. We then fit a free-ratio model, which assumes a different ratio (ω = *dN/dS*) for each branch in the tree. This produced a log likelihood value of *lf* = −9239311. The likelihood ratio test showed that the free-ratio model fits the data significantly better than the one-ratio model (*P*<0.001 for a χ^2^ distribution with df = 23). This indicates that the *dN/dS* ratios are indeed significantly different at different evolutionary stages and among lineages.

**Figure 3:**
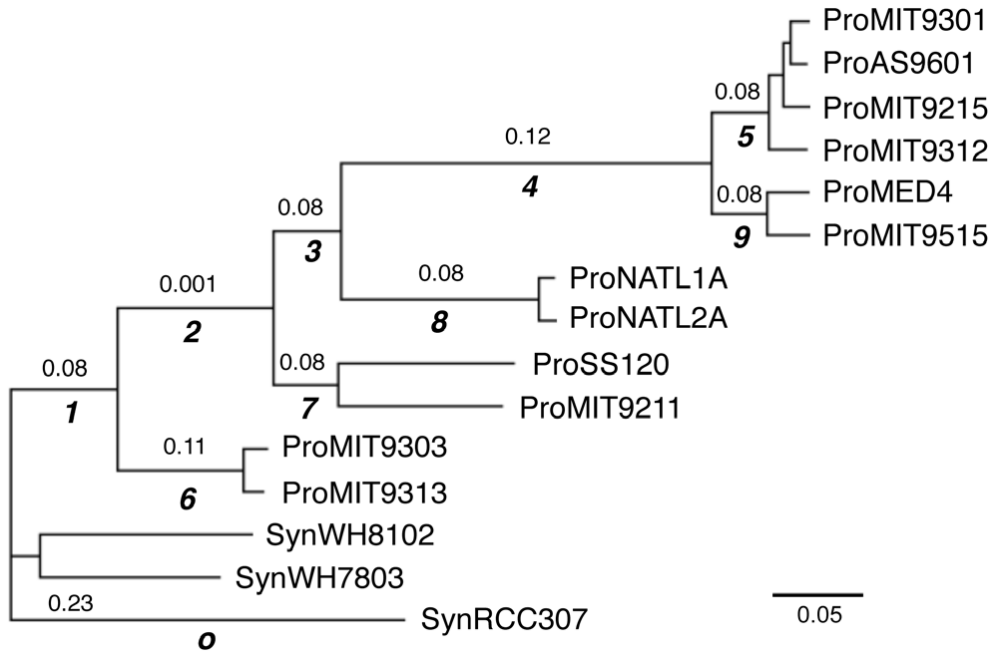
Maximum likelihood *dN/dS* ratios under the five-ratio-group-branch-model. The displayed phylogeny is an unrooted tree of 12 *Prochlorococcus* and 2 sister *Synechococcus* and an outgroup *Synechococcus* RCC307. The branches are drawn in proportion to their length. They are defined as the expected numbers of nucleotide substitutions per site, which is estimated from the concatenation of 100 conserved amino acid sequences by using the no-clock model. The tree topology, but not the branch lengths, is used to calculate the maximum likelihood *dN/dS* ratios. The ancestral branches are labeled below the branches (o: outgroup, 1–9). The maximum likelihood estimates of *dN/dS* ratios under a five-ratio-branch-model are reported on the corresponding branches. This model divides the tree into 5 ratio groups: ω_0_ = all *Synechococcus* branches; ω_1_ = branch #2; ω_2_ = branch #6 and *P.*MIT9303 clade; ω_3_ = branch #4; ω_4_ = *Prochlorococcus* background ratio (i.e., branch #1, branch #3, branch #7 and *P.*SS120 clade, branch #8 and *P.*NATL clade, branch #5, branch #9 and the entire *Prochlorococcus* HL clade). Branches for which no value is shown have the same ratios as their immediate ancestral branches.

Due to parameter rich nature of the free-ratio model, we obtained slightly different estimates of branch-specific *dN/dS* ratios between the replicate runs. Even so, they always show the same trend in ratio changes between the branches. Therefore, we designed a set of reduced branch-models and used a hypothesis testing strategy to verify the observed ratio changes across the tree. All the reduced models are described in Table 2 (the branch numbers refer to the labeled branches from the tree in Figure 3). The first two-ratio model (Model 2-1) assigns one ratio parameter to all the *Synechococcus* and one to all the *Prochlorococcus*. This model was compared with a one-ratio model to test whether *Prochlorococcus* has a significantly different ratio from *Synechococcus*. The second two-ratio model (Model 2-2) assumes that branch #1 (the ancestral branch of all the *Prochlorococcus*) has the same *dN/dS* ratio as *Synechococcus*, and it was compared with Model 2-1. If Model 2-1 has a higher likelihood value, then *Prochlorococcus* must have been under different selection pressure after its immediate split from *Synechococcus*. The first three-ratio model (Model 3-1) assigns a new ratio parameter for branch #2 (the ancestral branch of most *Prochlorococcus* after the split with the LL *P.* MIT9303 clade). Therefore, if it fits the data significantly better than the two-ratio models, branch #2 is most likely to have a distinct ratio from the other *Prochlorococcus* branches. The remaining two three-ratios models (Model 3-2, 3-3 and 3-4) are used to test if the descending branches have the same *dN/dS* ratio as branch #2 by comparing their likelihood values with Model 3-1. Both Model 4-1-1 & 4-1-2 are based on Model 3-1 but contain an extra parameter for either branch #4 that leads to the HL clade or for the entire HL clade and branch #4. They are designed to determine if branch #4 has a different ratio than other branches including its descending branches within the HL clade. Similarly, Model 4-2 contains an extra parameter for the *P.*MIT9303 clade based on Model 3-1. It is used to determine whether *dN/dS* ratios changed after the *P.*MIT9303 clade diversified from its *Prochlorococcus* ancestor.

**Table 2:** Log likelihood values and maximum likelihood estimates of *dN/dS* ratios under different branch models.

Ratio groups (ω = *dN/dS*)	NP	Log likelihood	ML estimates	
One ratio	ω1 = all branches	30	−9300717.05	ω1 = 0.092
2-1	ω1 = all *Synechococcus* branches	31	−9266700.48	ω1 = 0.226
	ω2 = all *Prochlorococcus* branches			ω2 = 0.067
2-2	ω1 = all *Synechococcus* branches+branch #1	31	−9268600.51	ω1 = 0.205
	ω2 = other *Prochlorococcus* branches			ω2 = 0.063
3-1	ω1 = all *Synechococcus* branches	32	−9245742.98	ω1 = 0.227
	ω2 = branch #2			ω2 = 0.001
	ω3 = other *Prochlorococcus* branches			ω3 = 0.080
3-2	ω1 = all *Synechococcus* branches	32	−9253199.03	ω1 = 0.228
	ω2 = branch #2+branch #7+*P.*SS120 clade			ω2 = 0.001
	ω3 = other *Prochlorococcus* branches			ω3 = 0.083
3-3	ω1 = all *Synechococcus* branches	32	−9258613.51	ω1 = 0.227
	ω2 = branch #2+branch #3+branch #8+*P.*NATL clade			ω2 = 0.024
	ω3 = other *Prochlorococcus* branches			ω3 = 0.078
3-4	ω1 = all *Synechococcus* branches	32	−9266619.31	ω1 = 0.226
	ω2 = branch #2+branch #3+branch #4+*P.*HL clade			ω2 = 0.065
	ω3 = other *Prochlorococcus* branches			ω3 = 0.070
4-1-(1)	ω1 = all *Synechococcus* branches	33	−9245422.25	ω1 = 0.227
	ω2 = branch #2			ω2 = 0.001
	ω3 = branch #4			ω3 = 0.117
	ω4 = other *Prochlorococcus* branches			ω4 = 0.080
4-1-(2)	ω1 = all *Synechococcus* branches	33	−9245720	ω1 = 0.227
	ω2 = branch #2			ω2 = 0.001
	ω3 = branch #4+*P.*HL clade			ω3 = 0.081
	ω4 = other *Prochlorococcus* branches			ω4 = 0.078
4-2	ω1 = all *Synechococcus* branches	33	−9245193.42	ω1 = 0.226
	ω2 = branch #2			ω2 = 0.001
	ω3 = branch #6+*P.*MIT9303 clade			ω3 = 0.105
	ω4 = other *Prochlorococcus* branches			ω4 = 0.077
5	ω1 = all *Synechococcus* branches	34	−9244816.89	ω1 = 0.226
	ω2 = branch #2			ω2 = 0.001
	ω3 = branch #6+*P.*MIT9303 clade			ω3 = 0.106
	ω4 = branch #4			ω4 = 0.118
	ω5 = other *Prochlorococcus* branches			ω5 = 0.076

We used both the Akaike Information Criterion (AIC) approach and the likelihood ratio test for model selection (Table 3). The results confirm our previous observations from the free-ratio model and are demonstrated in a final five-ratio-branch-model (Model 5 in Table 2 and Fig. 3). In summary, our results show: (1) *Prochlorococcus* branches always have lower genome-wide *dN/dS* ratios than marine *Synechococcus*, suggesting purifying selection has imposed stronger constraints on amino acid sequence evolution in *Prochlorococcus* than in marine *Synechococcus*, (2) there are consecutive decreases in the *dN/dS* ratio on the early ancestral branches of *Prochlorococcus* (branch #1 and branch #2). It's the same period when *Prochlorococcus* experienced major genome shrinkage, implying an association between intensified efficacy of purifying selection and massive gene deletions, (3) after the major genome reduction period, *dN/dS* ratios increased as the *Prochlorococcus* diversified and have remained relatively constant to this day, except for a slight decrease at branch #4 that leads to the HL clade.

**Table 3:** Model comparison and selection results.

*P*-value	ΔAIC	Better model	Conclusion	
2-1 vs. One-ratio	<10^−6^	−68031.14	2-1	*Prochlorococcus*≠*Synechococcus*
2-2 vs. 2-1	-	3800.06	2-1	branch #1≠*Synechococcus*
3-1 vs. 2-1	<10^−6^	−41913	3-1	branch #2≠other branches
3-2 vs. 3-1	-	14912.1	3-1	branch #7+*P.*SS120 clade≠branch #2
3-3 vs. 3-1	-	25741.06	3-1	branch #3 & #8+*P.*NATL clade≠branch #2
3-4 vs. 3-1	-	41752.66	3-1	branch #2 & #3 & #4+*P.*HL clade≠branch #2
4-1-(1) vs. 3-1	<10^−6^	−639.46	4-1-(1)	branch #4≠other branches
4-1-(1) vs. 4-1-(2)	-	−595.5	4-1-(1)	branch #4≠*P.* HL clade internal branches
4-2 vs. 3-1	<10^−6^	−1097.12	4-2	Branch #6+*P.*MIT9303 clade≠other branches

**NP**: number of parameters. Branch numbers refer to the branches from the tree in Figure 3.

***P-*** **value**: *P-*values of likelihood ratio tests.

**ΔAIC**: AIC (of the first model)- AIC (of the second model).

**Conclusion**: Decisions of a better model are based on the likelihood ratio test result (when parameter numbers are different) or on the Akaike Information Criterion (AIC = 2k -2ln (L), where k is the number of parameters in the model, and L is the maximized value of the likelihood function for the estimated model).

### *Prochlorococcus* lost genes have higher *dN/dS* ratios than retained genes

**Figure 4:**
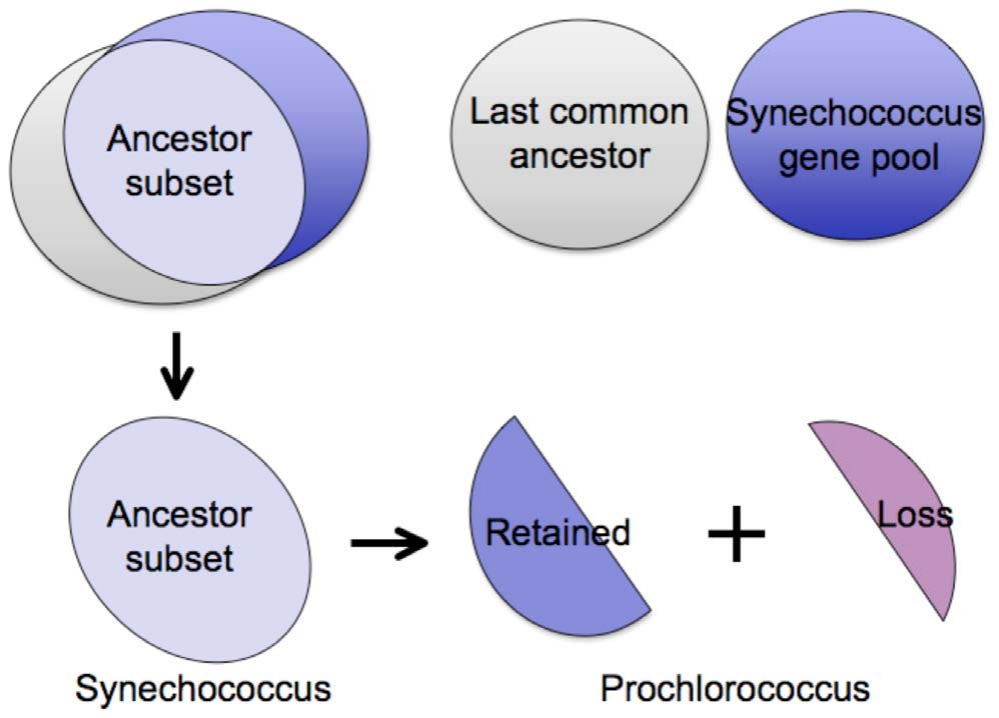
Ancestor-derived genes that were lost in the small *Prochlorococcus* but are retained in the extant *Synechococcus* genomes. A subset of 1641 ancestor-derived genes was identified from the 2028 genes in the reconstructed last common ancestor genome due to their presence in the 5 marine *Synechococcus* genomes. This subset is further divided into two groups: (1) the Lost group, which contains genes that are lost in the *Prochlorococcus* genomes; and (2) the Retained group, which contains genes that are kept in the *Prochlorococcus* genomes.

**Figure 5:**
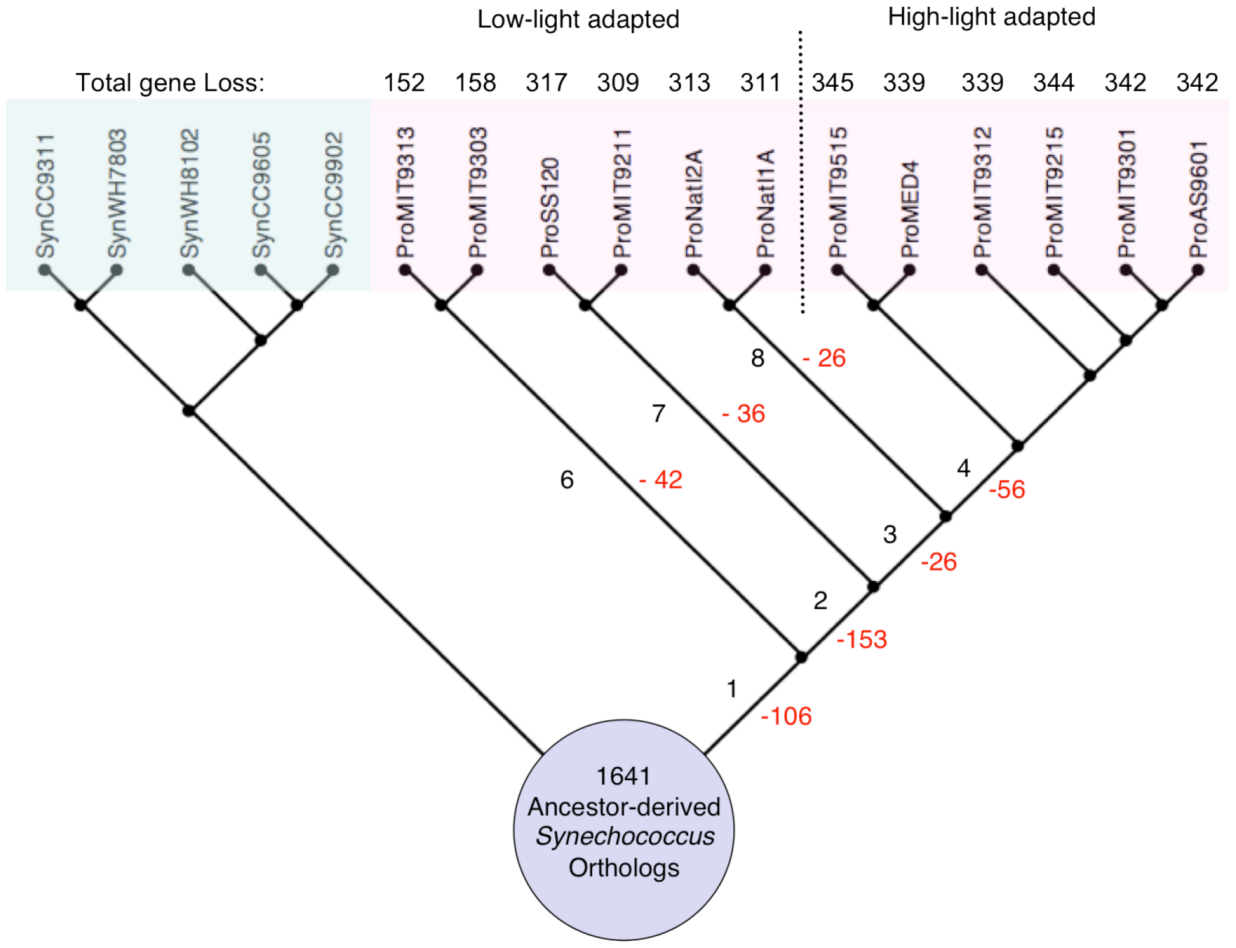
Step-wise *Prochlorococcus* gene losses reconstructed from the *Synechococcus* subset of ancestor-derived genes. Step-wise *Prochlorococcus* gene loss (red numbers) in the context of the subset of *Synechococcus* ancestor-derived genes is reported on the ancestor branches. The branch labels (black values) are the same as in Figure 3.

In order to determine the role of selection in the process of gene deletions, we measured two selection-related parameters (*dN/dS* ratio and the Codon Adaptation Index) for the ancestor-derived genes that are retained in the *Synechococcus* genomes but are lost in the *Prochlorococcus* on every internal branch of the *Prochlorococcus* phylogeny. Among the 2028 genes in the last common ancestor genome, 1641 have orthologs in all of the 5 in-group *Synechococcus* genomes. Therefore, we refer to them as the Ancestor-derived *Synechococcus* Orthologs (ASO)(Fig. 4). We then divided the 1641 ASO genes into two groups (the Lost group and the Retained group) based on the absence and presence of the genes in the reconstructed ancestor genome at every ancestor node of the tree. The numbers of the Lost and the Retained genes are summarized in Table 4. The step-wise gene losses in the *Prochlorococcus* evolution are demonstrated in Figure 5.

**Table 4:** Summary of gene loss and *dN/dS* ratios of the Loss and the Retained groups in the *Prochlorococcus* phylogeny.

Lost	Retained		*P*-value		
	Gene count	*dN/dS* (mean ± s.d.)	Gene count	*dN/dS* (mean ± s.d.)	
LCA	-	-	1641	0.115±0.084	NA
Branch #1	106	0.162±0.100	1535	0.111±0.081	<10^−5^
Branch #2	153	0.188±0.117	1382	0.103±0.072	<10^−6^
Branch #3	26	0.159±0.110	1356	0.102±0.070	0.014
Branch #4	56	0.149±0.088	1300	0.100±0.069	0.0001
Branch #6	42	0.123±0.064	1493	0.111±0.081	0.279
Branch #7	36	0.139±0.062	1346	0.104±0.072	0.002
Branch #8	26	0.113±0.054	1330	0.102±0.070	0.293
All	90	0.168±0.099	1185	0.097±0.066	<10^−6^

**Time**: LCA is the root of the tree, where is also the last common ancestor of *Prochlorococcus* and *Synechococcus*; Branch labels are same as those in Figure 3.

**Lost**: are genes that are missing in all the 12 extant *Prochlorococcus* genomes.

**Retained**: are genes that are present in all the extant *Prochlorococcus* genomes.

***P*** **-value**: *P*-values of Student's t tests. The null hypothesis is: the Lost group has the same mean as the Retained group

**Figure 6:**
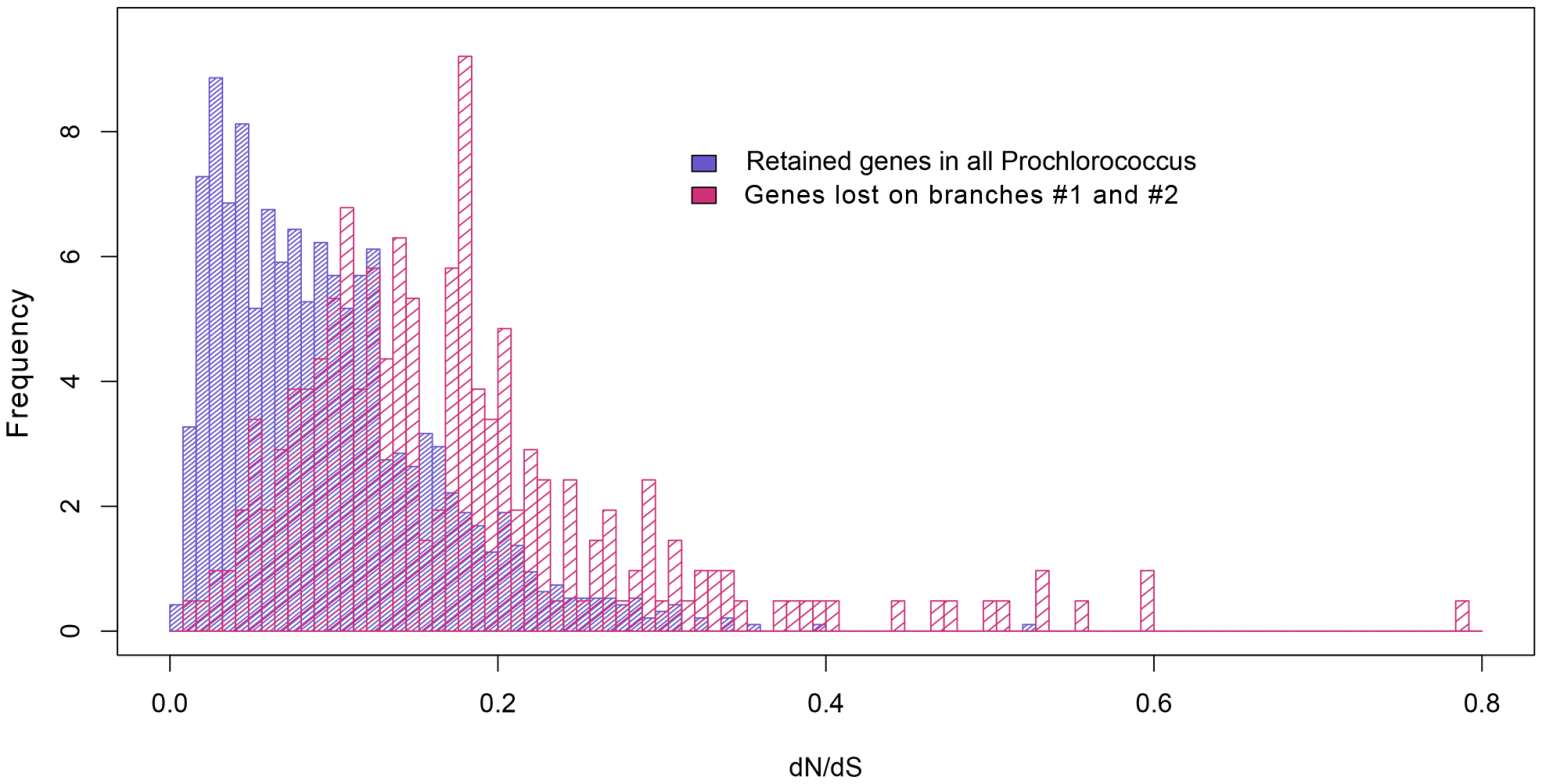
*dN/dS* distributions of the Lost and the Retained genes. The *dN/dS* ratios of a subset of the ancestral genes that are retained in the 12 extant *Prochlorococcus* and the 5 in-group marine *Synechococcus* genomes are shown in blue. The *dN/dS* ratios of a subset of the ancestral genes that are retained in the 5 in-group marine *Synechococcus* genomes, but were deleted in the *Prochlorococcus* during the early genome reduction period on branch #1 and branch #2 (branch numbers refer to the labeled branches in Fig. 3) are shown in red.

We first compared the *dN/dS* ratios of the Lost genes and the Retained genes. Genes with low *dN/dS* ratios (<1) are considered to be evolving under strong functional constraints and subject to strong purifying selection. The Lost group consistently shows a higher average *dN/dS* ratio than the Retained group (Table 4). In particular, the differences are highly significant on branch #1 and branch #2 during the genome reduction period (Student's t-test, *P-*value<10^−5^). More remarkably, genes that were lost during the early genome reduction period have significantly higher *dN/dS* ratios than those that were deleted in the later stages (Table 4. One-sided Student's t-test: Total gene loss on branch #1 and branch #2 vs. the Lost on branch #4, *P*-value = 0.021; Total loss on branch #1 and branch #2 vs. the Lost on branch #7, *P*-value = 0.002; Total loss on branch #1 and branch #2 vs. the Lost on branch #8, *P*<0.001; the Lost on branch #1 vs. the Lost on branch #6, *P-*value = 0.003). This result indicates that genes with the smallest fitness values were discarded first. Figure 6 shows the distinct distributions of the lost genes during the main period of genome reduction (the Lost genes on branch #1 and branch #2 combined) and of the genes that are retained in all the extant *Prochlorococcus* genomes. It again demonstrates that in general, genes of higher *dN/dS* ratios (of those <1), which are probably under relaxed selection pressure, were deleted in *Prochlorococcus* during the genome reduction period.

The Codon Adaptation Index (CAI) is useful for assessing how well genes are adapted to the local environment [40]. Due to the assumption that differences in the degree of codon usage bias largely reflect the differences in selective pressure on synonymous codons, the Codon Adaptation Index (CAI) is often positively correlated with the strength of selection. CAI values for the 1641 ancestor-derived genes were calculated for each of the 5 in-group marine *Synechococcus* genomes in the context of its genome-specific codon usage. The Lost group has significantly lower average CAI values compared to the Retained group except for branch #8 (Table 5). This supports our previous finding that genes under less stringent selection in the extant *Synechococcus* genomes were discarded early in *Prochlorococcus* evolution.

**Table 5:** Codon Adaptation Index of the Lost genes and the Retained genes in the marine *Synechococcus* genomes at different stages in *Prochlorococcus* evolution.

Lost	Retained		*P*-value		
	Gene count	CAI (mean ± std)	Gene count	CAI (mean ± std)	
***Synechococcus*** ** CC9605**					
LCA	-	-	1641	0.569±0.074	NA
Branch #1	106	0.551±0.063	1535	0.570±0.074	0.003
Branch #2	153	0.542±0.060	1382	0.574±0.075	<10^−6^
Branch #3	26	0.556±0.049	1356	0.574±0.076	0.075
Branch #4	56	0.520±0.082	1300	0.576±0.074	<10^−5^
Branch #6	42	0.542±0.060	1493	0.570±0.075	0.005
Branch #7	36	0.526±0.068	1346	0.575±0.076	0.0001
Branch #8	26	0.571±0.073	1330	0.575±0.075	0.801
***Synechococcus*** ** CC9902**					
LCA	-	-	1641	0.626±0.055	NA
Branch #1	106	0.612±0.061	1535	0.626±0.055	0.021
Branch #2	153	0.611±0.045	1382	0.628±0.056	<10^−4^
Branch #3	26	0.643±0.041	1356	0.628±0.056	0.075
Branch #4	56	0.591±0.067	1300	0.630±0.055	<10^−4^
Branch #6	42	0.597±0.049	1493	0.627±0.055	0.0003
Branch #7	36	0.589±0.062	1346	0.630±0.056	0.0004
Branch #8	26	0.623±0.038	1330	0.629±0.056	0.494
***Synechococcus*** ** WH8102**					
LCA	-	-	1641	0.572±0.075	NA
Branch #1	106	0.551±0.076	1535	0.574±0.074	0.003
Branch #2	153	0.543±0.062	1382	0.577±0.075	<10^−6^
Branch #3	26	0.546±0.066	1356	0.578±0.075	0.022
Branch #4	56	0.541±0.080	1300	0.580±0.074	0.0008
Branch #6	42	0.546±0.051	1493	0.574±0.075	0.002
Branch #7	36	0.525±0.057	1346	0.578±0.075	<10^−5^
Branch #8	26	0.581±0.081	1330	0.579±0.074	0.929
***Synechococcus*** ** CC9311**					
LCA	-	-	1641	0.676±0.046	NA
Branch #1	106	0.663±0.049	1535	0.677±0.046	0.007
Branch #2	153	0.662±0.046	1382	0.678±0.046	<10^−4^
Branch #3	26	0.667±0.052	1356	0.679±0.046	0.265
Branch #4	56	0.652±0.045	1300	0.680±0.045	<10^−4^
Branch #6	42	0.656±0.045	1493	0.677±0.046	0.004
Branch #7	36	0.650±0.038	1346	0.680±0.046	<10^−4^
Branch #8	26	0.680±0.034	1330	0.679±0.045	0.899
***Synechococcus*** ** WH7803**					
LCA	-	-	1641	0.603±0.069	NA
Branch #1	106	0.588±0.068	1535	0.604±0.069	0.020
Branch #2	153	0.584±0.067	1382	0.606±0.068	0.0002
Branch #3	26	0.587±0.063	1356	0.607±0.069	0.130
Branch #4	56	0.575±0.071	1300	0.608±0.068	0.001
Branch #6	42	0.575±0.045	1493	0.604±0.069	0.0002
Branch #7	36	0.572±0.059	1346	0.607±0.069	0.001
Branch #8	26	0.595±0.065	1330	0.607±0.068	0.347

**Time**: LCA is the root of the tree, which is also the last common ancestor of *Prochlorococcus* and *Synechococcus*; Branch labels are same as those in Figure 3.

***P*** **-value**: *P*-values of Student's t tests. The null hypothesis is: the Lost group has the same mean as the Retained group.

### Deleted gene functions do not suggest ecological specialization

**Figure 7:**
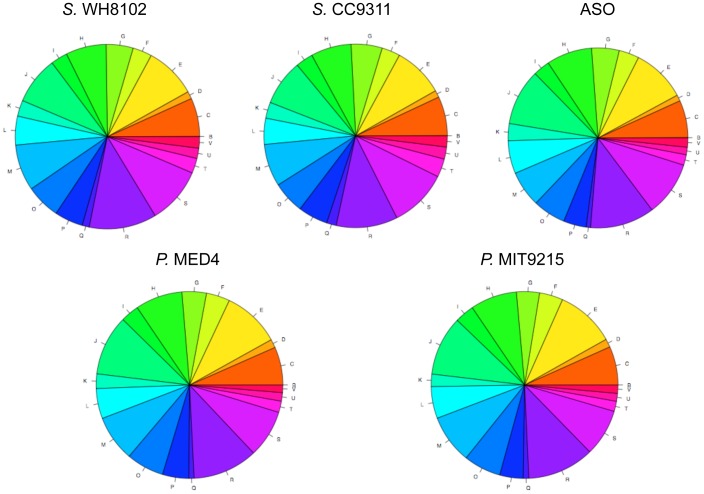
Functional profiles of representative marine *Synechococcus* and *Prochlorococcus* genomes, and of the ancestor-derived *Synechococcus* ortholog set. Functions were assigned to protein sequences that have significant hits to the Clusters of Orthologous Groups of proteins (COGs) annotation system in the Entrez Conserved Domain Database (CDD). A whole pie depicts the COG annotated gene pool of a genome. Each color represents a functional category of the COGs. The area under each color corresponds to the gene density in that COG category.

We employed the Clusters of Orthologous Groups of proteins (COGs) annotation system [41] to predict gene functions and to assess the functional effect of gene loss in *Prochlorococcus*. 1269 out of 1641 ancestor-derived genes (77.33%) were assigned to at least one COG category. The distribution of annotated genes in this ancestor-derived gene set is very similar to those of the individual *Synechococcus* genomes (Fig. 7). Moreover, the distributions of small *Prochlorococcus* genomes also resemble that of the ancestor gene set (Fig. 7), suggesting reduction in gene content didn't alter the general functional composition of *Prochlorococcus*.

**Figure 8:**
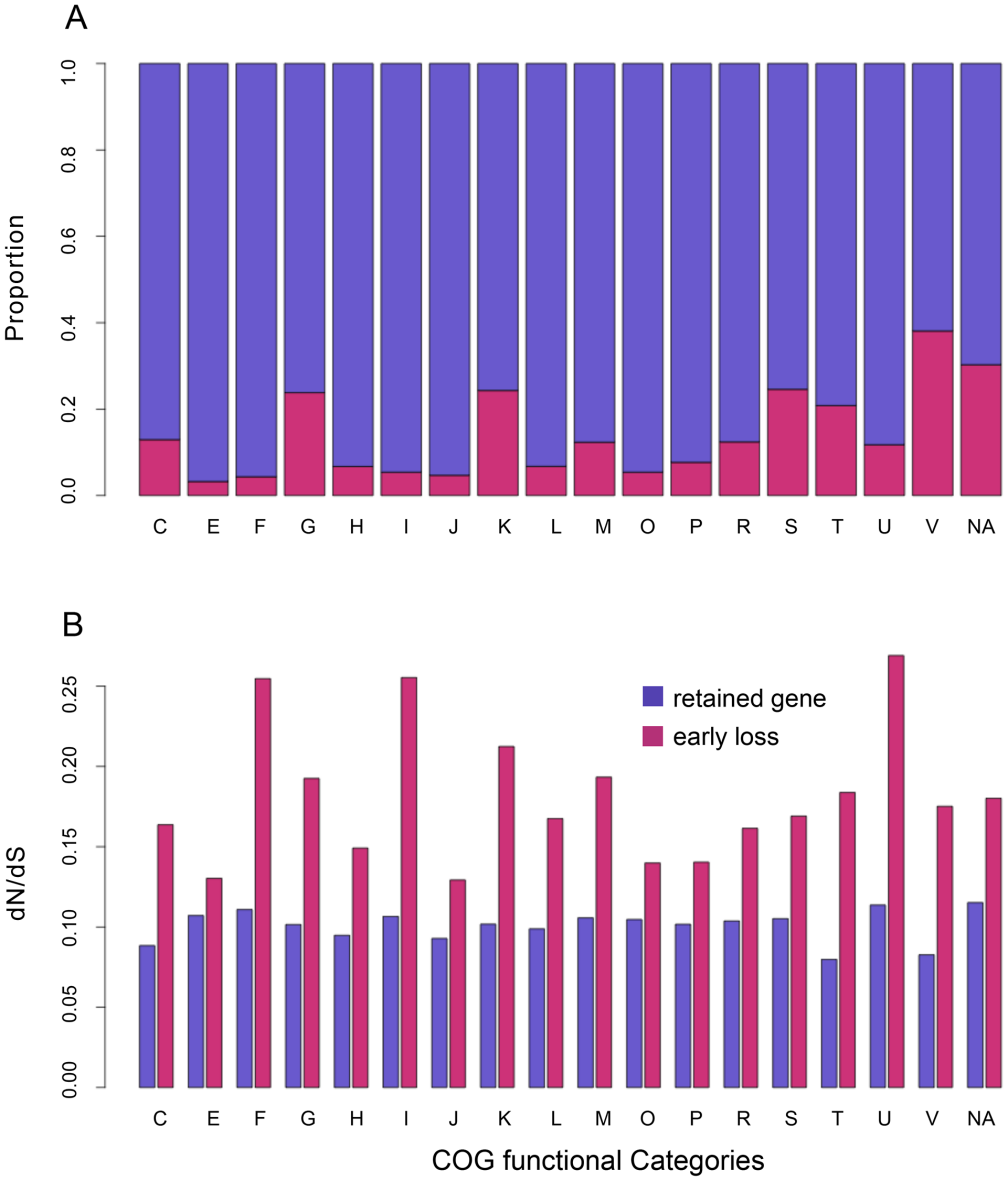
Functional distribution and *dN/dS* ratios of the Lost group during *Prochlorococcus* early genome reduction. **A**. The maroon area indicates the percentage of the genes that were deleted during the early genome reduction period (branch #1 and branch #2 in Fig. 3) in the ancestor-derived *Synechococcus* orthologs set (ASO). The purple area shows the percentage of the retained genes. COG abbreviations: C: Energy production and conversion; E: Amino acid transport and metabolism; F: Nucleotide transport and metabolism; G: Carbohydrate transport and metabolism; H: Coenzyme metabolism; I: Lipid metabolism; J: Translation, ribosomal structure and biogenesis; K: Transcription; L: DNA replication, recombination and repair; M: Cell envelope biogenesis, outer membrane; O: Posttranslational modification, protein turnover, chaperones; P: Inorganic ion transport and metabolism; R: general function prediction only; S: Function unknown; T: Signal transduction mechanisms; U: Intracellular trafficking and secretion; V: Defense mechanism; NA: no defined COG annotation. **B**. Bar plot of the average *dN/dS* ratios of the *Prochlorococcus* lost genes and the retained genes across functional groups. A subset of the ASO genes whose orthologs were lost in the *Prochlorococcus* during the early genome reduction period are shown in maroon. A subset of the ASO genes whose orthologs were kept in the *Prochlorococcus* genomes immediately after the early genome reduction, are shown in purple.

Among the 1269 COG annotated ancestor-derived genes, 259 were deleted during the early reduction period (on branch #1 and branch #2). These genes are distributed in all of the functional categories (Fig. 8A). Though some functional groups appear to have severer gene loss than the others, the deleted genes consistently have higher *dN/dS* ratio means and lower CAI means compared to the retained genes across all the function categories (Fig. 8B).

## Discussion

We demonstrated that (1) the small *Prochlorococcus* genomes were largely shaped by massive gene loss shortly after the split of *Prochlorococcus* and marine *Synechococcus*, (2) strong purifying selection occurred in *Prochlorococcus* during the genome reduction period, and (3) most *Prochlorococcus* lost genes have small fitness effects and are from nearly all functional categories.

Our results separate out the reduction process from the current state, thus decoupling genome reduction from current ecological niches. The small size and reductive characteristics of present *Prochlorococcus* genomes have been shaped by ancestral conditions before the radiation of most *Prochlorococcus* lineages, including the HL and LL groups. The forces driving *Prochlorococcus* genome reduction may not still be present or as strong. We suggest dividing *Prochlorococcus* evolutionary history into 3 sub stages: genome reduction, genome diversification (radiation of *Prochlorococcus*) and ecological niche specialization.

Functional analysis of lost genes at both early and recent evolutionary stages found no clear relationship between gene loss and ecological niche specialization. In contrast, extensive comparative analysis of extant *Prochlorococcus* genomes have identified a number of genes that are responsible for important ecological and physiological properties that differentiate *Prochlorococcus* ecotypes from each other [26]. Many of them are found to be concentrated in certain genomic regions called “genomic islands” [42]. These genomic regions are highly variable and are hot spots for gene gains [26], [42]. These observations suggest that horizontal gene transfer likely played a more important role than gene loss in ecological niche specialization and/or population diversification in *Prochlorococcus*. For instance, a group of phosphorus-uptake and metabolism genes have recently been found only in Atlantic *Prochlorococcus* populations where phosphorus concentrations are extremely low [36]. The limitation of phosphorus has imposed an ecosystem-specific selective pressure that has driven divergence between *Prochlorococcus* populations [36]. Again, the evolutionary forces responsible for recent *Prochlorococcus* diversification are probably not the same as what generated the reduced genomes at an earlier time.

Our results support the streamlining hypothesis [27], where selection for a more economical lifestyle has been the major driving force for genome reduction in *Prochlorococcus*. We are the first to estimate selection strength in a phylogenetic context and to demonstrate that the *Prochlorococcus* genome was subject to extremely strong purifying selection upon genome reduction. The genome-wide intensified selection was unlikely induced by changes of specific local environmental factors because altered environmental parameters usually only effect selection coefficients on a small subset of genes with particular functions. The *Prochlorococcus* genes that are lost during the early genome reduction period are from a variety of cellular processes spread across the genome and have higher *dN/dS* ratios (<1) regardless of function in *Synechococcus*. In spite of the large differences in gene content between *Prochlorococcus* and *Synechococcus* genomes, very few genes have been proposed to be responsible for ecological differences [27]. Therefore, the most plausible explanation is that *Prochlorococcus* had a very large effective population size during this time period and it imposed a strong purifying selection on the entire genome and subsequently led to the deletion of genes with higher *dN/dS* ratios.

Small effect genes are not necessarily discarded in large populations unless deletion of these genes confers selective advantage over maintaining them. This is true when the metabolic cost of maintaining small effect genes outweighs their potential benefits for the cell (e.g. allowing the cell to cope better with unfavorable conditions or environmental fluctuations). This scenario is most likely to occur in relative stable environments where nutrients are scarce (e.g., oligotrophic oceans) and competition for nutrients is severe. Under these conditions, advantages of a small genome—such as economics in energy and material for cell growth, maintenance and division [27]. increased surface area-to-volume ratios that improves nutrient uptake rate [10] and a reduction in self-shading [11] could be substantial enough that an economical lifestyle through genome reduction becomes a good strategy for ecological success.

It should be noted that not all deleted genes in the *Prochlorococcus* genome reduction are non-essential at individual levels, such as oxidative-stress genes, which are important for oxygen-producing marine cyanobacteria [43]. It's still unclear why such genes were also discarded from *Prochlorococcus*. One of the possible explanations is based on the Black Queen Hypothesis [37]. If coexisting organisms—e.g., marine *Synechococcus* –were capable of producing sufficient amounts of stress response enzymes to reduce reactive oxygen species to a level that was safe to all organisms in the community, then the related genes could become conditionally dispensable to *Prochlorococcus*.

**Figure 9:**
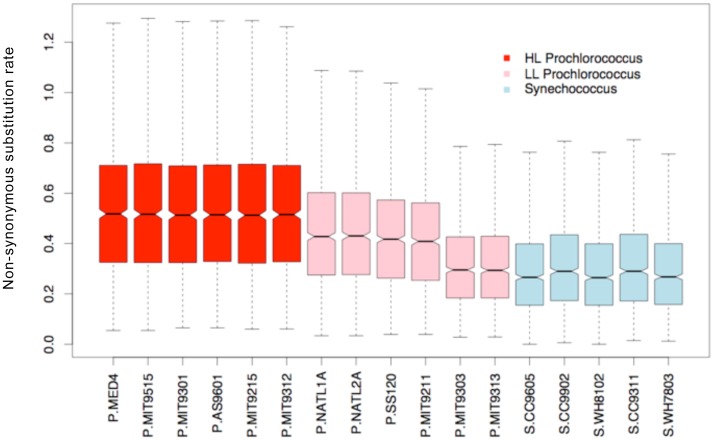
Variation in non-synonymous substitution rates across *Prochlorococcus* and *Synechococcus* genomes. All genomes were compared to an outgroup *Synechococcus* RCC307 to ensure the same divergence time. Red boxes: HL *Prochlorococcus*. Pink boxes: LL *Prochlrococcus*. Blue boxes: *Synechococcus*. The ends of the whiskers represent the lowest datum still within a 1.5 interquartile range (IQR) of the lower quartile, and the highest datum still within a 1.5 IQR of the upper quartile. A notch is drawn in each side of the boxes and non-overlapping notches of two plots indicate significantly different medians.

Our results did not rule out the possibility that an elevated global mutation rate might also have accompanied the *Prochlorococcus* genome size reduction. We found several non-essential DNA repair genes (Table 6) such as 6-O-methylguanine-DNA methyltransferase and DNA helicase RecQ that were deleted during the early genome reduction period and their loss could possibility cause a baseline mutation rate change. But the extremely low *dN/dS* ratio reveals that there was no accumulation of non-synonymous substitutions in comparison with synonymous substitutions. This is different than the case of the reduced endosymbiont genomes where an elevated mutation rate has been demonstrated to be responsible for the accumulation of deleterious mutations and subsequently led to exacerbated gene loss [44]. The lack of non-synonymous substitutions also means the mutation-driven model proposed by Marais et. al. [38], which is conditioned on fixation of small effect deleterious mutations does not apply to *Prochlorococcus* genome reduction. An accelerated protein evolution rate in *Prochlorococcus* in comparison with *Synechococcus* was previously reported from analyses of a small number of genomes [27], [32]. We also calculated the substitution rates at non-synonymous sites (*dN*) between individual in-group genomes to a common out-group genome *Synechococcus* RCC307. We found that the non-synonymous substitution rate not only increased from *Synechococcus* to *Prochlorococcus* but also significantly increased from low-light adapted to high-light adapted isolates within the *Prochlorococcus* group (Fig. 9). This indicates that the elevated protein evolution might not directly relate to genome reduction but rather associate with ecological niche specialization and diversification of *Prochlorococcus*.

**Table 6:** DNA repair genes lost during the early reduction period.

Genomic location in *Synechococcus* WH8102	Conserved Domain	Time of loss*	
ATP-dependent DNA ligase	+1315322…1317004	COG1793; Pfam01068; cd07897; cd07972	Branch #1
Methylated-DNA—protein-cysteine methyltransferase	−1617169…1617507	COG3693; Pfam01035; cd06445	Branch #2
RecQ, DNA helicase	−1864491…1866008	COG0514; Pfam00270; pfam00271	Branch #2
Nudix hydrolase	+1327195…1327626	COG1051; Pfam00293; cd04682	Branch #2
DNA/RNA helicase	−672895…674247	COG1061; Pfam04851; Pfam00176; Pfam00271; cd00046; cd00079;	Branch #2
DNA/RNA helicase	+2071435…2073954	COG1643; Pfam08482; Pfam04408;Pfam00270; Pfam00271; cd00046; TIGR01970;	Branch #2

*Branch numbers refer to the labeled branches from the tree in Figure 3.

*Prochlorococcus*, as an abundant marine photosynthetic group, is a focal point of climate change research. Do *Prochlorococcus* genomes have the genetic/evolutionary potential to cope with varying environment parameters? The answer is probably just as much as other microbes. Because of the large population sizes and ability to exchange genetic material, these genomes are unlikely to be evolutionary dead-ends. The current abundance and wide vertical distribution of diverse *Prochlorococcus* ecotypes in oligotrophic oceans has already demonstrated their evolutionary capabilities even with a small genome size. The growing collection of complete genome sequences and metagenomic data will provide additional opportunities to understand the population dynamics of *Prochlorococcus* and provide further insights into the evolutionary and ecological potential of this group of organisms.

## Materials and Methods

### Genome sequence data, gene prediction and annotation

The complete bacterial genome data set of our study contains 17 in-group cyanobacterial genomes (12 *Prochlorococcus*: *P.*MED4, *P.*MIT9515, *P.*AS901, *P.*MIT9301, *P.*MIT9215, *P.*MIT9312, *P.*NATL1A, *P.*NATL2A, *P.*SS120, *P.*MIT9215, *P.*MIT9313, *P.*MIT9303 and 5 marine *Synechococcus*: *S.*CC9605, *S.*CC9902, *S.*WH8102, *S.*CC9311, *S.*WH7803) and 31 other published cyanobacterial genomes. The genome sequences were downloaded from the Genome Assembly/Annotation Projects section on NCBI ftp://ftp.ncbi.nih.gov/genomes/Bacteria. To ensure gene annotations are consistent and comparable between the isolates we re-annotated the 17 in-group genomes and an outgroup *Synechococcus* genome RCC307 using the same gene prediction program GeneMarkS [45]. After translating DNA sequences to protein sequences, we determined gene function by searching predicted protein sequences against the Entrez Conserved Domain Database (CDD) of the Pfam [46] and Clusters of Orthologous Groups of proteins (COGs) [41] domain models using the Reverse Position-Specific BLAST algorithm (RPSBLAST) implemented in the NCBI BLAST search tool [47].

### Ancestral Gene Content Reconstruction and Genome Flux Analyses

(1) Generate an integrated multi-genome gene relationship table and identify common orthologous genes

We first used the protein-protein BLAST (blastp) algorithm to find highly similar protein sequences among the in-group genomes. Every predicted protein sequence of an in-group genome was blasted against a database of 48 cyanobacterial genomes. The inclusion of a large set of other published cyanobacterial genomes during blastp allows a low bit score cutoff threshold (40) to be used to generate a “top hit list” for each query. This still gives us high confidence in finding the real orthologs from the in-group genomes because we require the scores of the members of the in-group to be superior to the scores of other cyanobacterial genomes. A query gene can have at most one ortholog from each of the other in-group genomes. We collected such orthologous gene sets for each of the 17 in-group genomes, and then compiled them into a comprehensive multi-genome gene relationship table. This integrated gene table consists of two types of orthologous gene sets. The first type includes 1102 consistent orthologous gene sets whose homologous relationships have been identified congruently in all of the genome-specific tables. There are cases where an inconsistent orthologous gene set shares one or more genes with a consistent ortholog set. In these cases, the non-overlapping genes in the set are possible paralogs resulting from gene duplications in a subset of the genomes. We validate a paralog set by two criteria: (1) the same gene set is reported at least twice, and (2) every paralog in the set is confirmed by the blastp result of individual genome. Once confirmed, the paralogs are added to the related consistent ortholog set. The second part of the integrated gene table consists of consensus ortholog sets that are resolved from inconsistent but related gene sets. The most represented genes are chosen as the consensus orthologs from the possible paralogs based on the majority rule. The other paralogs are validated by the blastp results. 84 consensus ortholog sets were resolved by this method. In total we have identified 1186 orthologous gene sets of the 17 in-group genomes.

(2) Reconstruct the phylogeny of *Prochlorococcus* and *Synechococcus* using concatenated alignments of conserved orthologs

The pairwise *dN/dS* ratios were calculated for the orthologous gene sets using the yn00 program implemented in the Phylogenetic Analysis by Maximum Likelihood (PAML) software [39] (see Maximum likelihood analysis of sequence evolution rate below). Based on the *dN/dS* results, we selected 100 orthologous gene sets that (1) have the smallest means of pairwise *dN/dS* ratios, and (2) have average synonymous substitution rates in the range between the 1^st^ quartile (25%) and the 3^rd^ quartile (75%). Protein sequences of the selected orthologs were aligned individually in ClustalW2 [48]. The 100 alignments were then concatenated. The Gblocks [49] program was used to mask gapped and other noisy portions of the concatenated alignments, with a minimum block length of 10 amino acids and gaps allowed in any alignment position for no more than half of the sequences. Based on the concatenated alignment, we reconstructed a maximum likelihood tree using the Tree-PUZZLE program primed with a Neighbor-joining tree in the PHYLIP 3.6a [50]. The Jones-Taylor-Thornton (JTT) [51] amino acid substitution model was employed and all model related parameters were estimated from the data.

(3) Reconstruct the history of gene content evolution on the phylogenetic tree

We converted the integrated multi-genome gene relationship table, which was generated in the first step, into a R_(row)_×17_(column)_ character matrix of the gene state. In this matrix, each row is a homologous gene set and each column corresponds to an in-group genome. The value of every cell in the matrix indicates a genes current state and its copy number in one of the 17 in-group genomes. 0 means absent and any integer greater than 0 is the copy number. This character matrix was then mapped onto the phylogenetic tree, which we built in step 2. We applied two methods that are implemented in the Mesquite software [52] to reconstruct the ancestral gene states throughout the tree: (1) maximum parsimony, and (2) maximum likelihood. A single rate of changes between the states was assumed for both reconstruction methods. In the cases where a gene's state at the root cannot be unambiguously determined by the algorithms, we keep it in the last common ancestor only if this gene has at least one significant blastp hit from the out-group cyanobacteria. Genes with ambiguous predictions at the internal nodes are excluded from the downstream analyses. At each internal node a collection of genes with state N (N> = 1) were added to the ancestral genome at that evolutionary stage. Subsequently, a gene loss or gene gain event on an ancestral branch was determined by the change in quantity and direction of that gene's state between the two consecutive nodes.

### Maximum likelihood analysis of sequence evolution rates and *dN/dS* ratio

Protein sequences of the orthologs were aligned in the software ClustalW2 [48]. The corresponding nucleotide sequences were then mapped onto the protein alignment using custom Perl scripts to generate sets of aligned nucleotide sequences. Nucleotide substitution rates and *dN/dS* ratios were calculated using the maximum likelihood method as implemented in the Phylogenetic Analysis by Maximum Likelihood (PAML) software [39]. Pairwise *dN/dS* ratios were calculated from the synonymous and non-synonymous substitution rates, which were estimated by the yn00 program based on the HKY85 substitution model [53]. The codeml program was employed to compute branch-specific *dN/dS* ratios in a pre-determined phylogeny under different branch models. We used the topology of the concatenated gene tree. All model-related parameters (e.g., base frequencies and transition/transversion rate ratio) were estimated from the data. The likelihood value under each model was also reported by PAML. We then utilized the likelihood ratio test for model selection and hypothesis testing.

### Calculation of Codon Adaptation Index

We collected all of the ribosomal protein genes from each of the genomes and used them as reference sets to create genome-specific codon usage tables by the “cusp” program of the EMBOSS package [54]. The Codon Adaptation Index values were calculated by the “CAI” program of the EMBOSS package based on the codon usage tables.

## References

[1] Nakabachi A, Yamashita A, Toh H, Ishikawa H, Dunbar HE, et al (2006) The 160-Kilobase Genome of the Bacterial Endosymbiont Carsonella. Science (80-) 314: 267. 10.1126/science.1134196 17038615

[2] Schneiker S, Perlova O, Kaiser O, Gerth K, Alici A, et al (2007) Complete genome sequence of the myxobacterium Sorangium cellulosum. Nat Biotechnol 25: 1281–1289 Available: http://www.ncbi.nlm.nih.gov/pubmed/17965706. Accessed 13 September 2010. 17965706 10.1038/nbt1354

[3] Lynch M (2006) Streamlining and simplification of microbial genome architecture. Annu Rev Microbiol 60: 327–349. 16824010 10.1146/annurev.micro.60.080805.142300

[4] Xu L, Chen H, Hu X, Zhang R, Zhang Z, et al (2006) Average Gene Length Is Highly Conserved in Prokaryotes and Eukaryotes and Diverges Only Between the Two Kingdoms. Mol Biol Evol 23: 1107–1108. 16611645 10.1093/molbev/msk019

[5] Fraser CM, Gocayne JD, White O, Adams MD, Clayton RA, et al (1995) The minimal gene complement of Mycoplasma genitalium. Science 270: 397–403. 7569993 10.1126/science.270.5235.397

[6] Andersson JO, Andersson SG (1999) Genome degradation is an ongoing process in Rickettsia. Mol Biol Evol 16: 1178–1191. 10486973 10.1093/oxfordjournals.molbev.a026208

[7] Shigenobu S, Watanabe H, Hattori M, Sakaki Y, Ishikawa H (2000) Genome sequence of the endocellular bacterial symbiont of aphids Buchnera sp. APS. Nature 407: 81–86. 10993077 10.1038/35024074

[8] Akman L, Yamashita A, Watanabe H, Oshima K, Shiba T, et al (2002) Genome sequence of the endocellular obligate symbiont of tsetse flies, Wigglesworthia glossinidia. Nat Genet 32: 402–407. 12219091 10.1038/ng986

[9] Gil R, Silva FJ, Zientz E, Delmotte F, Gonzalez-Candelas F, et al (2003) The genome sequence of Blochmannia floridanus: Comparative analysis of reduced genomes. Proc Natl Acad Sci 100: 9388–9393. 12886019 10.1073/pnas.1533499100 PMC170928

[10] Giovannoni SJ, Tripp HJ, Givan S, Podar M, Vergin KL, et al (2005) Genome streamlining in a cosmopolitan oceanic bacterium. Science 309: 1242–1245 Available: http://www.ncbi.nlm.nih.gov/pubmed/16109880. 16109880 10.1126/science.1114057

[11] Dufresne A, Salanoubat M, Partensky F, Artiguenave F, Axmann IM, et al (2003) Genome sequence of the cyanobacterium Prochlorococcus marinus SS120, a nearly minimal oxyphototrophic genome. Proc Natl Acad Sci U S A 100: 10020–10025. 12917486 10.1073/pnas.1733211100 PMC187748

[12] Moran NA (2002) Microbial minimalism: genome reduction in bacterial pathogens. Cell 108: 583–586. 11893328 10.1016/s0092-8674(02)00665-7

[13] Andersson JO, Andersson SGE (2001) Pseudogenes, Junk DNA, and the Dynamics of Rickettsia Genomes. Mol Biol Evol 18: 829–839. 11319266 10.1093/oxfordjournals.molbev.a003864

[14] Gil R, Silva FJ, Pereto J, Moya A (2004) Determination of the core of a minimal bacterial gene set. Microbiol Mol Biol Rev 68: 518–537. 15353568 10.1128/MMBR.68.3.518-537.2004 PMC515251

[15] Moran NA, Mira A (2001) The process of genome shrinkage in the obligate symbiont Buchnera aphidicola. Genome Biol 2: RESEARCH0054. 11790257 10.1186/gb-2001-2-12-research0054 PMC64839

[16] Degnan PH, Lazarus AB, Wernegreen JJ (2005) Genome sequence of Blochmannia pennsylvanicus indicates parallel evolutionary trends among bacterial mutualists of insects. Genome Res 15: 1023–1033. 16077009 10.1101/gr.3771305 PMC1182215

[17] Tamas I, Klasson L, Canback B, Naslund AK, Eriksson AS, et al (2002) 50 million years of genomic stasis in endosymbiotic bacteria. Science (80-) 296: 2376–2379. 10.1126/science.1071278 12089438

[18] Ham RCvan, Kamerbeek J, Palacios C, Rausell C, Abascal F, et al (2003) Reductive genome evolution in Buchnera aphidicola. Proc Natl Acad Sci U S A 100: 581–586. 12522265 10.1073/pnas.0235981100 PMC141039

[19] Zinser ER, Schneider D, Blot M, Kolter R (2003) Bacterial evolution through the selective loss of beneficial Genes. Trade-offs in expression involving two loci. Genetics 164: 1271–1277. 12930738 10.1093/genetics/164.4.1271 PMC1462639

[20] Perez-Brocal V, Gil R, Ramos S, Lamelas A, Postigo M, et al (2006) A Small Microbial Genome: The End of a Long Symbiotic Relationship? Science (80-) 314: 312–313. 10.1126/science.1130441 17038625

[21] Partensky F, Hess WR, Vaulot D (1999) Prochlorococcus, a marine photosynthetic prokaryote of global significance. Microbiol Mol Biol Rev 63: 106–127. 10066832 10.1128/mmbr.63.1.106-127.1999 PMC98958

[22] Moore LR, Rocap G, Chisholm SW (1998) Physiology and molecular phylogeny of coexisting Prochlorococcus ecotypes. Nature 393: 464–467. 9624000 10.1038/30965

[23] Partensky F, Blanchot J, Vaulot D (1999) Differential distribution and ecology of Prochlorococcus and Synechococcus in oceanic waters: a review. Marine cyanobacteria, eds Charpy L, Larkum A (Musée Océanographique, Monaco) 19: 457–475.

[24] Moore LR, Chisholm SW (1999) Photophysiology of the Marine Cyanobacterium Prochlorococcus: Ecotypic Differences among Cultured Isolates. Limnol Oceanogr 44: 628–638 Available: http://www.jstor.org/stable/2670673.

[25] Johnson ZI, Zinser ER, Coe A, McNulty NP, Woodward EM, et al (2006) Niche partitioning among Prochlorococcus ecotypes along ocean-scale environmental gradients. Science 311: 1737–1740. 16556835 10.1126/science.1118052

[26] Kettler GC, Martiny AC, Huang K, Zucker J, Coleman ML, et al (2007) Patterns and implications of gene gain and loss in the evolution of Prochlorococcus. PLoS Genet 3: e231. 18159947 10.1371/journal.pgen.0030231 PMC2151091

[27] Dufresne A, Garczarek L, Partensky F (2005) Accelerated evolution associated with genome reduction in a free-living prokaryote. Genome Biol 6: R14. 15693943 10.1186/gb-2005-6-2-r14 PMC551534

[28] Luo H, Friedman R, Tang J, Hughes AL (2011) Genome reduction by deletion of paralogs in the marine cyanobacterium Prochlorococcus. Mol Biol Evol 28: 2751–2760. 21531921 10.1093/molbev/msr081 PMC3203624

[29] Rocap G, Larimer FW, Lamerdin J, Malfatti S, Chain P, et al (2003) Genome divergence in two Prochlorococcus ecotypes reflects oceanic niche differentiation. Nature 424: 1042–1047. 12917642 10.1038/nature01947

[30] Palenik B, Ren Q, Dupont CL, Myers GS, Heidelberg JF, et al (2006) Genome sequence of Synechococcus CC9311: Insights into adaptation to a coastal environment. Proc Natl Acad Sci 103: 13555–13559. 16938853 10.1073/pnas.0602963103 PMC1569201

[31] Dufresne A, Ostrowski M, Scanlan DJ, Garczarek L, Mazard S, et al (2008) Unraveling the genomic mosaic of a ubiquitous genus of marine cyanobacteria. Genome Biol 9: R90. 18507822 10.1186/gb-2008-9-5-r90 PMC2441476

[32] Hu J, Blanchard JL (2009) Environmental sequence data from the Sargasso Sea reveal that the characteristics of genome reduction in Prochlorococcus are not a harbinger for an escalation in genetic drift. Mol Biol Evol 26: 5–13. 18845550 10.1093/molbev/msn217

[33] Ahlgren NA, Rocap G, Chisholm SW (2006) Measurement of Prochlorococcus ecotypes using real-time polymerase chain reaction reveals different abundances of genotypes with similar light physiologies. Environ Microbiol 8: 441–454. 16478451 10.1111/j.1462-2920.2005.00910.x

[34] Sullivan MB, Waterbury JB, Chisholm SW (2003) Cyanophages infecting the oceanic cyanobacterium Prochlorococcus. Nature 424: 1047–1051. 12944965 10.1038/nature01929

[35] Sullivan MB, Coleman ML, Weigele P, Rohwer F, Chisholm SW (2005) Three Prochlorococcus cyanophage genomes: signature features and ecological interpretations. PLoS Biol 3: e144. 15828858 10.1371/journal.pbio.0030144 PMC1079782

[36] Coleman ML, Chisholm SW (2010) Ecosystem-specific selection pressures revealed through comparative population genomics. Proc Natl Acad Sci U S A 107: 18634–18639. 20937887 10.1073/pnas.1009480107 PMC2972931

[37] Morris JJ, Lenski RE, Zinser ER (2012) The Black Queen Hypothesis: evolution of dependencies through adaptive gene loss. MBio 3: e00036–12–. 22448042 10.1128/mBio.00036-12 PMC3315703

[38] Marais G, Calteau A, Tenaillon O (2008) Mutation rate and genome reduction in endosymbiotic and free-living bacteria. Genetica 10.1007/s10709-007-9226-6 18046510

[39] Yang Z (2007) PAML 4: phylogenetic analysis by maximum likelihood. Mol Biol Evol 24: 1586–1591. 17483113 10.1093/molbev/msm088

[40] Sharp PM, Li W-H (1987) The codon adaptation index-a measure of directional synonymous codon usage bias, and its potential applications. Nucleic Acids Res 15: 1281–1295. 3547335 10.1093/nar/15.3.1281 PMC340524

[41] Tatusov RL, Fedorova ND, Jackson JD, Jacobs AR, Kiryutin B, et al (2003) The COG database: an updated version includes eukaryotes. BMC Bioinformatics 4: 41. 12969510 10.1186/1471-2105-4-41 PMC222959

[42] Coleman ML, Sullivan MB, Martiny AC, Steglich C, Barry K, et al (2006) Genomic islands and the ecology and evolution of Prochlorococcus. Science 311: 1768–1770. 16556843 10.1126/science.1122050

[43] Scanlan DJ, Ostrowski M, Mazard S, Dufresne A, Garczarek L, et al (2009) Ecological Genomics of Marine Picocyanobacteria. Microbiol Mol Biol Rev 73: 249–299. 19487728 10.1128/MMBR.00035-08 PMC2698417

[44] Moran NA, McLaughlin HJ, Sorek R (2009) The Dynamics and Time Scale of Ongoing Genomic Erosion in Symbiotic Bacteria. Science (80-) 323: 379–382. 10.1126/science.1167140 19150844

[45] Besemer J, Lomsadze A, Borodovsky M (2001) GeneMarkS: a self-training method for prediction of gene starts in microbial genomes. Implications for finding sequence motifs in regulatory regions. Nucleic Acids Res 29: 2607–2618. 11410670 10.1093/nar/29.12.2607 PMC55746

[46] Finn RD, Mistry J, Tate J, Coggill P, Heger A, et al (2010) The Pfam protein families database. Nucleic Acids Res 38: D211–22. 19920124 10.1093/nar/gkp985 PMC2808889

[47] Altschul SF, Gish W, Miller W, Myers EW, Lipman DJ (1990) Basic local alignment search tool. J Mol Biol 215: 403–410. 2231712 10.1016/S0022-2836(05)80360-2

[48] Larkin MA, Blackshields G, Brown NP, Chenna R, McGettigan PA, et al (2007) Clustal W and Clustal X version 2.0. Bioinformatics 23: 2947–2948. 17846036 10.1093/bioinformatics/btm404

[49] Castresana J (2000) Selection of conserved blocks from multiple alignments for their use in phylogenetic analysis. Mol Biol Evol 17: 540–552. 10742046 10.1093/oxfordjournals.molbev.a026334

[50] Felsenstein J (1989) PHYLIP - Phylogeny Inference Package (Version 3.2). Cladistics 5: 164–166.

[51] Jones DT, Taylor WR, Thornton JM (1992) The rapid generation of mutation data matrices from protein sequences. Comput Appl Biosci 8: 275–282. 1633570 10.1093/bioinformatics/8.3.275

[52] Maddison WP, Maddison DR (2007) Mesquite: a modular system for evolutionary analysis. Version 2.0 http://mesquiteproject.org.

[53] Hasegawa M, Kishino H, Yano T (1985) Dating of the human-ape splitting by a molecular clock of mitochondrial DNA. J Mol Evol 22: 160–174 Available: http://dx.doi.org/10.1007/BF02101694. 3934395 10.1007/BF02101694

[54] Rice P, Longden I, Bleasby A (2000) EMBOSS: the European Molecular Biology Open Software Suite. Trends Genet 16: 276–277. 10827456 10.1016/s0168-9525(00)02024-2

